# The realities of HIV prevention. A closer look at facilitators and challenges faced by HIV prevention programmes in Sudan and Yemen

**DOI:** 10.1080/16549716.2019.1659098

**Published:** 2019-09-09

**Authors:** Fatima Bashir, Maha Ba Wazir, Barbara Schumann, Kristina Lindvall

**Affiliations:** Department of Epidemiology and Global Health, Umeå University, Umeå, Sweden

**Keywords:** HIV, prevention, Sudan, Yemen

## Abstract

**Background**: HIV/AIDS prevention has historically encountered many obstacles. Understanding the factors affecting HIV/AIDS prevention is central to designing and implementing suitable context-specific interventions. Research relating to HIV prevention in the Middle East and North African region is required to address the gradually increasing HIV epidemic.

**Objective**: This study aimed to explore the perspectives of employees/health care professionals who are working or have worked within HIV prevention in Sudan and Yemen on the challenges and facilitating factors facing HIV prevention.

**Methods**: A qualitative approach was employed using an open-ended questionnaire. Sixteen stakeholders from governmental and non-governmental agencies participated in the study. The questionnaire focused on the various challenges and facilitating factors facing HIV prevention as well as proposed possible solutions from the perspectives of the participants. The data were analysed using thematic analysis.

**Results**: The study illustrated the similarities in context and HIV prevention systems between Sudan and Yemen. Thematic analysis resulted in three main themes: I) much is achieved despite difficulties; II) a programme left to be paralysed; this theme addressed the main obstacles facing HIV prevention in Sudan and Yemen generating a total of six sub-themes; III) comprehensive change is needed. The participants drew focus and attention to vital changes required to improve the delivery of HIV prevention services.

**Conclusion**: Increased financial support for HIV prevention in Sudan and Yemen is urgently needed. De-stigmatisation and increased political support, advocacy and improved legislation for people living with HIV (PLHIV) are required for the sustainability and effectiveness of HIV prevention programmes in Sudan and Yemen. Civil society organisations must be aided and supported in their role in engaging key populations.

## Background

The human immunodeficiency virus (HIV) epidemic was first recognised in the 1980s, and within two decades, the virus had spread to over 190 countries [1,2]. Internationally, the epidemic rapidly took precedence, and in 1996, the United Nations formed the Joint United Nations Programme on HIV/AIDS (UNAIDS), whose primary concern was a global HIV response [3].

Despite this global attention, HIV/AIDS persists as one of the leading causes of fatality in Africa, with an incidence rate of approximately two million cases/year [4]. While the introduction of anti-retroviral therapy (ART) has significantly delayed the progression of the epidemic, the unmitigated annual incidence suggests that biomedical prevention alone will not suffice, thereby prompting the need to address HIV/AIDS prevention from a behavioural perspective. Five main key population groups, who are the main targets of prevention programmes, have been identified by the World Health Organisation; these include men who have sex with men (MSM), sex workers, injectable drug users, people confined in prison and other closed settings, and transgender people [5]. The WHO also identified groups that might be particularly vulnerable in specific countries or settings: migrant workers, refugees and, of particular importance, young women. The social and cultural challenges faced by these vulnerable groups contribute to their vulnerability, but also to a lack of data regarding HIV/AIDS prevalence among them.

According to UNAIDS, approximately 36 million individuals worldwide live with HIV, 70% of which are in Africa, and in 2017 almost 1 million people died of AIDS-related illness worldwide [4]. The consequential global response to the HIV epidemic has resulted in a reduction in global prevalence to less than 1%, thereby reducing new HIV infections by 47% since the peak of the epidemic in 1996 [6,7]. However, this relative success was marked by numerous challenges. Many researchers have investigated limiting factors for HIV prevention programmes. For example, in many settings, it is not enough to encourage prevention by condoms without addressing the sensitive subject of religion and abstinence [8]. In other countries, the very mention of MSM is taboo, creating a wall of resistance from political leaders and decision makers.

The Middle East and North African region (MENA) is comprised of approximately 24 countries including Sudan and Yemen (Table 1); over 91% of MENA countries are predominantly Muslim [9]. Despite the global trend of declining HIV incidence, the MENA region as well as Eastern Europe and Central Asia (EECA) have witnessed an increase in newly diagnosed HIV cases in recent years. Since 2001, the number of new HIV infections in the MENA region has increased by 31%, the highest increase among all the regions in the world [10].

The epidemic in MENA is not considered as generalised [11], with a prevalence of 0.1% [10]. However, a paucity of HIV-related data may have created a false representation of a low HIV epidemic in the region. Existing data on HIV in MENA may not accurately portray the true incidence due to a possible underestimation resulting from the poor quality and non-representative surveillance systems of most Ministries of Health in the region. In many countries, underreporting might be a direct result of war and conflict. Only three countries (Morocco, Djibouti and Iran) have a relatively well-functioning surveillance system. By the end of 2015, the known number of people living with HIV (PLHIV) in all MENA countries was significantly lower than their estimated national figures [11,12]. Saba et al. conducted a wide study characterising the progress in HIV research in the MENA region; only 1.2% of the records and data obtained were related to MENA, a finding that further echoes the poor contribution of MENA to the global HIV literature [12,13]. However, despite this paucity in data from the region, the low HIV prevalence has been seen by many as a justification to halt further progression of the epidemic [14].

Sudan and Yemen are no exception when it comes to the deficiency of data in the region. Despite the observed progress in surveillance systems, the literature on HIV/AIDS is scanty [15,16]. According to national estimates, the prevalence of HIV in Sudan is approximately 0.3% [15]. However, a recent meta-analysis showed an overall pooled HIV prevalence of 1%, suggesting that HIV prevalence may be highly underestimated [17]. Similarly, in Yemen, despite a very low prevalence of HIV among the general population, it is suggested that the political insecurity and war in recent years has affected the notification of new HIV cases [16]. Moreover, the number of AIDS-related deaths has risen significantly as a result of poor access to anti-retroviral drugs (ARVs) [18].

The political instability of these two countries poses a barrier to combatting HIV. Furthermore, the cultural and social environment of both countries poses a special challenge in conducting behavioural and risk assessments. The religious atmosphere in both predominantly Muslim countries creates a difficult task for prevention programmes targeting those who participate in high risk sexual activities that are prohibited by religion and in some cases by law. This has led to a gap in interventions addressing behavioural changes that may conflict with the countries’ religious and cultural values. Therefore, this study aimed to explore the challenges and facilitating factors that HIV prevention programmes are facing in Sudan and Yemen from the perspective of representatives of HIV agencies.

## Methods

### Study design

This study utilised a qualitative study design with an open-ended questionnaire as the data collection tool. A qualitative method was chosen to provide a holistic picture and detailed knowledge about the perspectives of the participants regarding the challenges and facilitating factors faced by HIV prevention programmes [19].

The initial study plan was to conduct semi-structured interviews via Skype or telephone. However, this was not feasible due poor internet and landline connections. Therefore, a written questionnaire was chosen for data collection. The questionnaire was developed based on the first and second author’s prior knowledge of the setting and the available national and international literature on HIV prevention in both Sudan and Yemen. Prior to sending out the questionnaire, this was also discussed between all co-authors and revised accordingly. The final questionnaire consisted of eleven open-ended questions, designed to have narrative written answers, and focused on the challenges and facilitating factors that the employees faced in their line of work within HIV prevention [20]. The questions primarily covered the following areas: the type of HIV response (behavioural, medical or both), the obstacles encountered and their consequences, the facilitating factors aiding their efforts, the difficulties with key populations and how these obstacles were addressed (Appendix 1).

### Data collection

Data were collected from March 2017 to August 2017. Potential participants in Sudan were contacted by FB and potential participants in Yemen by MB. The participants were contacted first by telephone and then by email by the two first authors. The initial participants were contacted through the respective Ministry of Health of each country. Purposive sampling [21] was used to recruit participants from both governmental and non-governmental organisations who could share their knowledge and experience of working within HIV prevention [22]. This was combined with snowball sampling, where participants who were contacted first introduced us to other potential participants [23]. The aim of this sampling was to include a variety of opinions from different perspectives; therefore, participants were selected from different HIV clinics/centres with different backgrounds and work experience in HIV prevention [22]. The frame of sampling was based on two main criteria:

1. Being employed (current or past) by national HIV prevention programmes or a non-governmental organisation working with HIV.
2. At least 5 years of work experience in an HIV prevention programme.

A total of 26 individuals were invited to answer the questionnaire. Both genders were included and participants (both current and previous employees) were of a variety of backgrounds in diverse positions within their respective organisations. The questionnaire was sent to all participants via e-mail, in both Arabic and English; the participants were able to select their preferred language. Reminder emails were sent (maximum of three times) and any clarification requested by the participants was promptly answered. Responses in Arabic were translated into English by the first two authors (FB and MB) and subsequently reviewed by both researchers. The desired number of participants was sixteen, an approximate number of responses judged by the researchers to provide saturation when analysing the data, and included eight participants from each country.

### Data analysis

All questionnaires were analysed using thematic analysis. The two first authors led the analysis. Inductive thematic analysis was used in this study; the researchers started the analysis with an open mind as to what themes could emerge from coding. The flexibility of thematic analysis allowed the researchers to consider both manifest and latent content in data analysis. Analysis was conducted following the six steps mentioned by Braun and Clarke, beginning with familiarising the data, followed by generating initial codes, searching for themes, reviewing themes, defining and naming themes and finally producing the final report. Initially, the responses were analysed individually by each of the two first authors then negotiated between them and the initial codes were generated [24–26] (Table 4). The preliminary analysis and results were subsequently discussed and negotiated between all four authors. The analysis was facilitated using Open Code 4.03 software.

### Ethical considerations

The study was carried out in accordance with the principles of the 1975 Helsinki Declaration. Participation was voluntary and written consent was obtained from all participants prior to answering the questionnaire. Participant confidentiality was highly prioritised and thoroughly maintained; only the research team had access to the data. All results were documented and recorded without any possibility of tracing individual informants.

## Results

The total number of participants who responded was sixteen, with eight from each country (six females and ten males). The number of participants working/worked for NGOs was ten, while the number of those working/worked for national governmental HIV prevention programmes in both countries was six participants (Table 5).

Overall, the participants were forthcoming with their answers, providing elaborated responses to most questions. The thematic analysis resulted in three main themes that paint a comprehensive picture of HIV prevention in Sudan and Yemen. The themes were: I) much is achieved despite difficulties; this theme generated a total of three sub-themes; II) a programme left to be paralysed; this theme put the spotlight on different obstacles faced by HIV prevention, i.e. religious, political and financial among others, which create a challenging working environment that often counteracts the efforts made; six sub-themes were generated and; III) comprehensive change is needed; here, the participants discuss the most important ways to overcome the challenges associated with the law, politics, finances or communities such as increased funding and a focus on the de-stigmatisation of HIV/AIDS (Figure 1).

### Much is achieved despite difficulties

This theme describes the available national responses to HIV/AIDS within prevention programmes in both countries, according to the work experience of our participants. This theme sheds light on the nature of the HIV response in Sudan and Yemen in order to better understand how this response may be affected by different factors. Three sub-themes resulted, depending on the type and the recipients of these services: improving community prevention, strengthening health care provider prevention and providing support HIV/AIDS treatment and care.

Community prevention in both Sudan and Yemen is directed to the general public in general and to key populations, particularly MSM and female sex workers (FSW). The main activities included awareness raising, behavioural change particularly among target groups, enabling supportive environments and providing support for PLHIV.

*‘The HIV prevention programme in general focuses on: behaviour change communication (BCC), stigma reduction, HIV testing and counselling (HTC), HIV prevention among key populations and vulnerable groups, prevention of mother-to-child transmission of HIV (PMTCT) services, sexually transmitted infection (STI) prevention and control, condom programming’ (p4 – Sudan)*.

‘Increase HIV awareness using faith-based and religious leaders and capacity building, health and community systems strengthening, maintain and enhance an enabling environment and increase political commitments. Endorsement of the law to protect the community from HIV infection and protect PLHIV rights in 2009’ (p2 – Yemen).

Many participants shed light on the importance of HIV prevention within the health care sector. This included the development of guidelines for universal safety precaution, post-exposure prophylaxis (PEP) and training and capacity building for health care workers. All participants mentioned HIV treatment and care in a broad sense; however, more than half of them mentioned ART specifically. An important aspect of treatment and care is the establishment of well-functioning referral systems for confirmed cases, especially among expecting mothers. One staff member of the National Programme in Yemen stated:

‘Guidelines development on universal safety precaution and capacity building of health care providers in all Yemen on the guidelines. Establishment of a referral system for HIV infected mothers to HIV treatment and care services’ (p1 – Yemen).

Most of the participants reported that most HIV prevention services cover the entire country. However, a few participants stated that this was not always the case. When answering which geographical areas were covered and why they were chosen, participants provided different responses and reasoning. An NGO worker from Sudan explained why some areas were excluded from the programme:

‘I think the area of Butana, Gulaelnahul localities are excluded from the programme, sometimes according to the limitation of budget and stigma or the power of community leaders who refuse to talk about HIV/AIDS or STIs in public and consider that a taboo’ (p15 – Sudan).

### A programme left to be paralysed

Several participants elaborated their answers on the challenges and facilitating factors they encountered, while others provided broad non-detailed answers. The sub-themes that were generated were: deficient HIV funding is a constant obstacle, insufficient data and surveillance on HIV, HIV prevention is confronted with many logistic challenges, the political climate hinders work on HIV prevention, an unfavourable religious context and the social and cultural environment imposes many restrictions on HIV prevention.

#### Deficient HIV funding is a constant obstacle

Participants from both countries stated that HIV prevention programmes depend chiefly on external donor support. Lack of funds and limited resources has led to the unsustainability, interruption and/or termination of HIV programmes which, in turn, have generated a loss of trust, particularly from vulnerable key populations.

‘Lack of sustainability of funds … lack of sustainability in services affected the reliability of prevention services by creating demand among affected people without accessible services’ (p10 – Sudan).

‘The national AIDS control programme implements prevention interventions when there is fund from donors, otherwise the programme is paralysed and implemented by some NGOs through awareness raising’ (p1 – Yemen).

#### Insufficient data and surveillance on HIV

Participants reported that the scarcity of HIV-related data and conducted research has resulted in planning of HIV prevention programmes based on estimations of prevalence. Stigmatisation and criminalisation of key populations appeared to be the primary reason for withholding HIV status information and for the reluctance in participating in surveys. Participants from Sudan describe a problematic setting in which HIV-related studies may be affected or even terminated by police and security forces. One NGO staff member from Sudan stated:

‘M*any studies are arranged, but sometimes the study is withheld due to security and/or police stopping it, not only studies among FSW and/or MSM, but even studies among university students, armed forces etc.’ (p4 – Sudan)*

#### HIV prevention is confronted with many logistic challenges

Many logistic challenges were raised by participants which directly affected their line of work. These included lack of accessibility of HIV services, difficulties in reaching target groups, a disjoint and disconnect between sexually transmitted infection (STI) prevention/management and HIV prevention, difficulties in designing/implementing of interventions due to inaccurate data and high turnover of human resources.

‘STI management is an entirely separate component to the HIV programme and there is hardly any linkage between HIV and STI programmes. A significant opportunity is lost since STIs could be used as entry points for the HIV programme’ (p7 – Yemen).

The participants reflected on the interrelated factors of political instability, culture and religion which have also inevitably led to many of these logistic challenges. For example, the lack of political commitment towards the rights of PLHIV and their criminalisation, which is based on religious teachings, has led to a lack of data and inaccessibility of target groups. The stigma and cultural/religious barriers further extend to those working in the field who are viewed as encouraging unwanted behaviour, thereby creating a sense of insecurity.

‘Stigma and discrimination against the target groups (MSM and FSW) due to religious and social norms, a negative effect: encouraging high risk groups to practice sex by using safe sex which is against our norms and traditions’ (p5 – Yemen).

The unstable political climate in both countries has given rise to a vulnerable population of refugees and internally displaced people (IDPs). Participants reflected on the difficulties they consistently face when working with refugees and IDPs, although the condition is much more acute in Yemen, considering recent political tensions and violent conflicts in the country. The supporting role of NGOs and civil society organisations (CSOs) was considered as a facilitating factor by several participants. NGOs and CSOs have a major role in identifying and reaching key populations and a call for increased support for these organisations is quite evident.

#### The political climate hinders work on HIV prevention

Most participants stated similar political challenges in both countries despite different degrees of acuteness; in Yemen, the recent war has created a more urgent precedence. Legislation and laws that criminalise key populations stand as a hurdle to reach out to target groups. Moreover, many participants mentioned that the denial of HIV by policymakers and disregard of HIV as a priority have weakened the response to HIV. This threat is often extended to HIV programme personnel, creating an unsafe environment for the conduct of prevention programmes. This was reiterated by many of our participants, i.e. those working with governmental programmes and NGOs, who feel this has become a serious impediment to their work:

‘We met very huge challenges regarding the policies and security obstacles, we cannot do anything unless go back to the Ministry of Health and have permission from the security force’ (p9 – Sudan).

‘Security personnel threats (threat of arrest, surveillance of programmes and interventions). Security holds the view that the nature of our work contributes to the spread of HIV, and think that we encourage sexual practices, all because we work with the most-at-risk groups’ (p12 – Yemen).

On the other hand, a few participants from both countries recognised the gradually increasing political commitment despite the lack of resources.

#### Unfavourable religious context

A recurrent reflection discussed was the role of religion; many participants believe the incriminating nature of Islamic law may hinder HIV/AIDS preventive measures. This was apparent in the case of condom distribution where it was looked upon as encouraging unacceptable behaviour such as homosexuality and sexual promiscuity, which are considered foreign to Muslim societies. Conversely, a few participants recognised the progressive support of HIV/AIDS prevention among many religious clerics. Condom distribution has become gradually accepted, although it may be confined to the constraints of family planning. While this view does not advocate directly for condoms as a means of reducing HIV transmission, this critical step is viewed by participants as a facilitating factor for their HIV prevention efforts. One NGO staff member described this as being a ‘major blow’ to their efforts:

‘Being a Muslim country and with a general trend towards Islamisation of everything, there are some voices that keep on calling for an Islamic strategy to address the disease. The main issue is that this turns the terminology around to be more and more incriminating (e.g. FSW are prostitutes; MSM are homosexuals etc.). If this continues to build up it will constitute a major blow to the response in Sudan’ (p4 – Sudan).

‘Denial in some states, believing that the disease is coming from outside and that we, as Muslims, do not have THIS kind of behaviour’ (p4 – Sudan).

#### The social and cultural environment imposes many restrictions for HIV prevention

Stigma and discrimination were the most consistently mentioned obstacles among all participants, in addition to gender-based discrimination against women; this has created an impediment against the uptake of HIV prevention services. An NGO worker from Sudan referred to this gender-based discrimination highlighting the tolerance received for men and not women:

‘HIV-infected women often face additional stigma, as HIV infection is associated with promiscuity, which is generally tolerated for men, but not accepted for women’ (p4 – Sudan).

‘Some men do not tell their wives that they are HIV-positive and do not take them to hospital or to HIV counselling centres to check and make sure if she is infected (male-dominated society). Women are considered as something they own, and her freedom and dignity are seized’ (p12 – Yemen).

Participants reported that stigma and discrimination against PLHIV not only exist in the community but also within the health care sector. An NGO worker from Yemen drew attention to this aspect saying:

‘The doctor also refuses to provide health services such as a surgical intervention for PLHIV on the pretext of protecting himself from virus transmission. At the same time, he does not take into account occupational safety standards when providing surgical services to people or other patients who do not know that they are HIV-infected or do not inform him that they are infected by HIV or other viruses’ (p12 – Yemen).

### Comprehensive change is needed

Suggested solutions that the participants described as being necessary to overcome the challenges that HIV prevention programmes encountered in both countries were similar and included increasing funds, increasing political commitment, the de-stigmatisation of HIV/AIDS, improved legislation/protection of the rights of PLHIV, support and enhancement of the role of civil society organisations and lastly improving surveillance systems.

‘The most important solutions can be … Make a change to some laws, put pressure on decision-makers, create forums to fight stigma, support civil society organisations working with the most vulnerable groups’ (p12 – Yemen).

‘Increasing the funds for HIV/AIDS programmes, the federal Ministry of Health has to adopt a clear HIV/AIDS policy to improve the work environment, conduct more advocacy sessions for security officers and other stakeholders, update intervention approach and strategy’ (p9 – Sudan).

Finally, we asked the participants to grade the performance of HIV/AIDS prevention programmes in their respective countries from 0 to 10, given that 0 = total failure and 10 = total success. The average score given by the participants was 6.5 out of 10. For each country separately, the average grades given by our participants grading the HIV/AIDS prevention efforts in Yemen was 7 and in Sudan was 6. These grades reflect the perspectives of the participants on the effectiveness of the design, planning and implementation of HIV/AIDS prevention programmes in Sudan and Yemen.

## Discussion

This study aimed to explore the perspectives of employees working or who have previously worked in HIV/AIDS prevention in Sudan and Yemen on the challenges and facilitating factors they encountered during their work and their outlooks on possible solutions to these challenges. The analysis resulted in three main themes: much is achieved despite difficulties; a programme left to be paralysed, a theme highlighting the various challenges faced by HIV prevention; and comprehensive change is needed. Participants from both Sudan and Yemen provided similar responses, suggesting the similarity of the contexts between the two countries. Since participants focused on the challenges facing HIV prevention, the discussion of findings will weigh more heavily on this sector. These challenges have summoned increasing attention by policy makers and researchers, and through understanding why and how they operate, it may be possible to design and develop more suitable interventions compatible with the ultimate goals of HIV prevention programmes and the narrating context in which they are implemented.

### HIV responses in Sudan and Yemen

In Sudan, most of the HIV-related activities are orchestrated by the Sudan National AIDS and STI control Programme (SNAP) in close partnership with local and international NGOs. HIV prevention programmes cover the whole country; however, they are concentrated in certain locations according to available epidemiological data, which dictate the degree of active implementation of prevention programmes. Sudan is classified as a concentrated HIV epidemic country, where the HIV epidemic has spread rapidly among one or more subpopulations but is not well-established in the general population, with the highest HIV prevalence in the Eastern zone including the Kassala and Red Sea states [15]. HIV prevention is focused on behavioural change and targets key populations. The main HIV prevention activities include HIV testing and counselling, prevention of mother-to-child transmission (PMTCT), STI prevention and control, condom programming, blood safety/universal precautions, infection control in health care settings, stigma reduction and knowledge and behavioural change and communication.

In Yemen, the National AIDS Programme (NAP), in collaboration with local/international NGOs and CSOs, carries out many activities similar to those undertaken in Sudan, including awareness raising, HIV testing and counselling, PMTCT, condom promotion and distribution. The programme runs in all 22 governorates, but services vary and are concentrated in specific locations based on many factors such as population census, epidemic profile, ease of service provision and presence of vulnerable key populations [16].

### Challenges facing HIV responses

Participants agreed that the sexual and behavioural culture surrounding HIV/AIDS is a crucial aspect of HIV spread and prevention which demands increased attention when implementing preventive measures. Stigma and shame were identified as leading disincentives facing HIV/AIDS prevention efforts. In most countries in the MENA region, religious and conservative norms prevail, Sudan and Yemen being no exception. HIV/AIDS is strongly linked to practices deemed prohibited and unacceptable to cultural and religious norms in the region, thereby further accentuating the stigma associated with HIV. Much of the stigma is rooted in the misinformation and general lack of knowledge of HIV transmission. A study by Mohamed et al. revealed that, while many of the Sudanese general public are aware of the disease, significant misconceptions on HIV transmission were prevalent [27]. Similar findings of misconception among MSM were reported in Yemen by Mirzazadeh et al. regarding comprehensive knowledge of HIV in addition to poor levels of knowledge of HIV transmission [28].

A crucial element of stigma raised by many participants was the stigma endured by PLHIV from health care professionals which has also been supported in the findings of previous studies [29,30]. A study conducted by the Kaiser Family Foundation reported that 15% of gay and bisexual men, who make up over a quarter of all new HIV infections in the United States, experienced stigma and unfair treatment from medical personnel and 30% of are less likely to discuss HIV related concerns with health care providers [31]. The effects of social stigma are further extended to staff members and employees of HIV prevention programmes. Many experience threats and hostile behaviour [15], as stated by our participants, not only from society but from armed forces and religious leaders, as it is widely perceived that HIV prevention serves to embolden and encourage illegal sexual activity. This has in turn led to a high degree of staff overturn and loss of valuable employees.

Both Sudan and Yemen are considered low income countries with high rates of poverty. An inevitable consequence is the widespread expansion of the sex trade, which has led many women, and often men, into this vicious cycle as in many neighbouring countries in MENA and in sub-Saharan Africa (SSA) [32]. Participants identified sex workers as one of the major target groups that is notoriously difficult to engage with. The aggressive social hostility towards sex workers and MSM renders them unable to seek professional health care or counselling for fear of being abused, harassed or perhaps legally persecuted [33]. This study also highlights the facilitating role of CSOs in engaging key populations. Despite being limited in number and capacity, CSOs have proven to be integral to HIV prevention efforts in Sudan and Yemen, as in many countries in SSA [34].

The participants reported religious obstacles as a consistent difficulty. The Islamic religion prohibits any form of extramarital sex and homosexuality; both are prohibited sins that may be punishable by law [35]. Islamic countries did not previously consider the HIV/AIDS epidemic invasion a threat as their communities were assumed to be protected by Islamic practice [36]. However, the current situation is contrary to their beliefs; the MENA region is home to the fastest growing HIV/AIDS rates globally [9]. Furthermore, the main mode of transmission of HIV in Sudan and Yemen is through unprotected heterosexual intercourse [28,37]. While both Sudan and Yemen are two of the few countries to apply Sharia law to homosexuality, it is not mentioned explicitly in their respective legislations. Rather, other laws are used to criminalise acts of homosexuality and punish perpetrators [38].

In most countries in the MENA region, including Sudan and Yemen, the constitution is largely based on the teachings of Sharia law [39] and policymaking is often determined and steered by holy scriptures. This constricts the limits at which policies may be implemented to aid HIV/AIDS prevention. The law in Sudan and Yemen criminalises drug use, extramarital sex [28] and homosexuality, all of which are strongly penalised [38]. In as many as 47 countries, similar laws have been used to persecute PLHIV, in some cases for offences as meagre as spitting [40].

The absence and/or inconsistency of political commitment towards HIV/AIDS prevention is a concern voiced loudly by the participants. Similar findings have been reported by Kelly et al.; 30% of NGOs in Africa reported lack of political support as a major impeding factor for HIV prevention [41]. Despite this, our findings suggest a slow but gradual, significant improvement and increase in political support and advocacy for HIV prevention [15]. Many voices are calling for governments to address these legal impediments [33]. In Yemen, progressive laws were successfully enacted by the Yemeni Parliament in 2009 [42]. In Sudan, however, attempts to provide legislative and policy support for PLHIV is still ongoing and the draft of legislation supporting PLHIV is yet to be endorsed [15]. Ministries such as the Ministry of Social Welfare and the Ministry of Justice have taken great strides in ensuring HIV-sensitive social protection for PLHIV in Sudan, including nutritional support and vocational training, although key populations (sex workers, gay men and other men who have sex with men, people who inject drugs, transgender people, prisoners) are not recognised as key beneficiaries [43].

Lack of funding for HIV/AIDS prevention composes a constant hurdle. Kelly et al. reported similar findings; 70–100% of NGOs in Africa, the Caribbean and Eastern/Central Europe identified low funding as a leading barrier to HIV prevention [41]. The Global Fund to Fight AIDS, Tuberculosis and Malaria is a private-public partnership focusing on allocating resources to combat HIV globally [44]. Many national development programmes in both Sudan and Yemen are funded by the Global Fund, either partially or completely, with limited contributions from the government. In recent light of the humanitarian crisis in the two countries, HIV/AIDS has lost its precedence and many interventions have been terminated. In Yemen, the majority of the country’s expenditure has been redirected to relieve the humanitarian crisis and the remaining HIV prevention activities since 2011 have been largely funded by the Global Fund (75% of total HIV funding) [16]. The HIV response in Sudan has been well-financed by international aid (62% of total HIV spending), primarily from the Global Fund; however, this contribution has significantly declined (from 14.5 in 2011 to 9.1 million USD in 2013) [45]. Domestic funding for the national AIDS response similarly declined from 6.6 to 3.7 million USD in the period from 2011 to 2013 [45].

Data collection and HIV surveillance are frequently discussed challenges. Many factors have been attributed to this: the extreme stigmatisation of key groups, fear of criminal persecution and the problematic task of monitoring high-risk populations such as refugees and IDPs. While surveillance in the MENA region has advanced significantly, Sudan and Yemen are listed as two of the countries with partially functioning HIV surveillance systems [11].

When questioned on plausible solutions from their perspectives, the responses from participants of both countries were similar and were partly reflected in the average performance scores they allocated to the HIV prevention programmes in their respective countries. Many solutions were suggested; increased funding being the first and most common, followed by de-stigmatisation, increased political commitment, protective legislation and support for PLHIV. In recognition of the importance of CSOs, a common suggestion was the increased support for CSOs and local NGOs. Finally, our participants reiterated the importance of further research and improved surveillance systems in the two countries, a recommendation emphasised in many previous studies in the region [11].

### Methodological considerations

A qualitative approach was utilised to obtain a clearer view and in-depth perspective on aspects of HIV prevention that may not be captured by a quantitative approach from the perspectives of those working in various HIV prevention programmes in Sudan and Yemen. To increase the trustworthiness of the study, measures were taken to affirm credibility, dependability and transferability. The inductive approach of coding in addition to the coherent, systematic analysis of data ensured that all data were accurately accounted for and represented. We thus regard the results as having a high level of credibility and reflecting well the intended focus of the study. To further enhance the credibility of our study, the participants were chosen based on their experience in the field of HIV prevention in both countries, from both governmental and non-governmental agencies, and who therefore could provide in-depth information with regards to HIV prevention programmes in Sudan and Yemen. We believe this diverse pool of participants extended additional dependability to our results [19]. None of the researchers have any prior acquaintance with the participants and no personal gain or interests were involved. The information provided by the participants was not influenced in any way or manner and was initially thoroughly interpreted independently by each researcher and the findings were then developed by oscillating between the resulting interpretations.

Although generalisation was not our intention for the study, the striking similarities seen in both the Sudanese and Yemeni contexts suggest that our findings may be applicable in other conservative Islamic conservative contexts throughout MENA. We believe the same findings would have been found had the study been repeated in the same context with the same participants, and in that, we regard the results as having a high level of transferability. The initial study design incorporated in-depth semi structured live and/or internet-based interviews of our participants who are located in Sudan and Yemen. As this was not feasible, a questionnaire of open-ended questions was utilised instead. This created a limitation of the study as there was no room for probing and following up questions that would have benefitted from further elaboration and clarification. Another limitation worth mentioning is the inadequate representation from the Sudanese National Programme, which might create a potential bias in the representativeness of participants.

### Conclusion and recommendations

The obstacles facing HIV prevention in Sudan and Yemen are very substantial and are amalgamated and overlapping in a very complex manner. However, financial obstacles, stigma and discrimination were the leading difficulties encountered. The role of society/culture, political constraints and religious norms similarly had a large influence on the implementation of HIV prevention programmes. Further studies are needed to address stigma and discrimination in religious conservative settings to better inform future policies and interventions. The low prevalence of HIV in Sudan and Yemen provides an opportunity for action that should not be bypassed; however, the need for a more contextualised approach for HIV prevention in MENA is clearly visible and an urgent response is required. Increasing engagement with academic sectors could provide an insight on possible solutions on policy development, implementation and evaluation. The improvement of legislation and human rights of PLHIV in Sudan and Yemen, although progressing, require urgent attention. Finally, faith-based approaches are viable routes to HIV prevention in MENA that should be explored further.

## Paper context

HIV/AIDS prevention in the MENA region faces many hindering factors. Understanding these obstacles is vital when designing suitable context-specific interventions. However, research on HIV/AIDS within MENA is sparse. This study aimed to explore the perspectives of those who are working of who have worked with HIV prevention programmes in Sudan and Yemen on the difficulties they have encountered in their respective countries. Equipped with this knowledge, interventions may be tailored to the needs of each context to produce more effective outcomes.
