# Supplementary material for: The realities of HIV prevention. A closer look at facilitators and challenges faced by HIV prevention programmes in Sudan and Yemen
Source: Glob Health Action. 2019 Sep 9;12(1):1659098. doi: 10.1080/16549716.2019.1659098 (PMC6746302; doi:10.1080/16549716.2019.1659098)
Supplement: Supplemental Material [file ZGHA_A_1659098_SM8485.docx]

**Table 1:** List of MENA Countries according to UNAIDS (1)

| Afghanistan | Algeria | Bahrain |
| --- | --- | --- |
| Djibouti | Egypt | Iran |
| Iraq | Jordan | Kuwait |
| Lebanon | Libya | Morocco |
| Oman | Pakistan | Occupied Palestinian Territory |
| Qatar | Saudi Arabia | Somalia |
| Sudan | South Sudan | Syria |
| Tunisia | United Arab Emirates | Yemen |

**Table 2:** General Information of the two study settings

|  | Republic of Sudan | Republic of Yemen |
| --- | --- | --- |
| Surface Area | 728,215 sq mi^*^ | 194,553 sq mi |
| Population | 40,235,000 | 25,408,000 |
| Country Capital | Khartoum | San’aa until 2014, Aden since 2015 |
| Language | Arabic | Arabic, South Semitic languages (e.g. Mehri and Soqotri) |
| Religion | Islam/Christian minority | Islam/ Christian and Jewish minorities |
| GDP per Capita | $4,578 | $2,476 |

sq mi^*^ square mile.

**Table 3:** HIV in Sudan and Yemen

|  | Sudan | Yemen |
| --- | --- | --- |
| HIV Prevention programme | SNAP^*^ 1987 | NAP^**^ 1987 |
| Funding | Mainly from Global Fund to Fight AIDS, Tuberculosis and Malaria | Mainly from Global Fund to Fight AIDS, Tuberculosis and Malaria |
| Estimated HIV prevalence | 0.3-0.4% (2) | 0.2% (4) |
| Estimated HIV prevalence among high risk groups | FSW^+^ 1.3%  MSM^++^ 1.4% (3) | FSW 1.23%  MSM 5.9% (4) |

SNAP^*^ Sudan National AIDS & STI Programme (AIDS; Acquired Immunity Disease Syndrome, STI; Sexually Transmitted Infections), NAP^**^ National AIDS Programme, FSW^+^ female sex workers,

MSM^++^ Men who have sex with men.

**Table 4:** Sample of coding process using thematic analysis

| Text | Codes | Sub-theme | Theme |
| --- | --- | --- | --- |
| In Sudan, as in most countries in the region and the world, HIV/AIDS is surrounded by strong stigma and discrimination, as many people have misconceptions about HIV transmission and fear of being infected through daily-life contacts with HIV infected persons | Stigma      Discrimination      Misconception about HIV transmission | The social and cultural environment imposes many restrictions for HIV prevention | A prevention programme left to be paralysed |
| Being a Muslim country, and with a general trend towards Islamisation of everything, there are some voices that keep on calling for Islamic strategy to address the disease. The main issue is that this turns the terminology around to be more and more incriminating (e.g. FSW are prostitutes; MSM are homosexuals etc.). If this continues to build up it will constitute a major blow to the response in Sudan. | call for Islamic strategies  Islamisation of response increases discrimination  Islamisation may damage the response | Unfavourable religious context | A prevention program left to be paralyzed |

**Table 5:** Characteristics of study participants

| Participant No. | Country | Gender of Participants | Organization | Professional Background | Years of experience within HIV |
| --- | --- | --- | --- | --- | --- |
| 1 | Yemen | Female | National Programme | Medical doctor | >10 |
| 2 | Yemen | Female | NGO/National Programme | Medical physician | >15 |
| 3 | Sudan | Male | NGO | Community Development, project management | >15 |
| 4 | Sudan | Male | NGO | Medical officer | >10 |
| 5 | Yemen | Male | National Programme | Public Health Professional | >15 |
| 6 | Yemen | Female | National Programme | Pediatrician | >10 |
| 7 | Yemen | Male | National Programme | Pharmacist | >10 |
| 8 | Sudan | Male | NGO | Psychologist | >10 |
| 9 | Sudan | Male | NGO | Political scientist | >10 |
| 10 | Sudan | Male | NGO | Public and Tropical Health Specialist | >15 |
| 11 | Sudan | Male | NGO | Sociologist | >20 |
| 12 | Yemen | Male | NGO | National trainer in HIV prevention programmes | >15 |
| 13 | Yemen | Female | NGO | Teacher | >10 |
| 14 | Sudan | Female | National Programme | Psychologist | >15 |
| 15 | Sudan | Female | NGO | Social Scientist | >10 |
| 16 | Yemen | Male | National Programme | - | - |

**Figure 1:** The main themes and sub-themes of the thematic analysis

**Appendix 1:**

**Questionnaire about HIV Prevention Programme**

**General Information:**

Gender…………………

Name of Employing Organization: …………………………………………………………………………

Position at Organization: ……………………………………………………………………………………….

Professional Background ……………………………………………………………………………………….

Start date of work in HIV Prevention Programme (most recent) ………………………………………

End date of work with HIV Prevention Programme if applicable …………………………………….

Job title/ Profession in HIV Prevention Programme……………………………………………………….

1-How long have you been working in HIV/AIDS prevention programme (both previous and current)?

2-What is/was your main tasks and activities within the HIV/AIDS Prevention Programme and in which geographical area?

3-Can you give a general description about the services offered by the HIV/AIDS Prevention Programme?

4-a) What are/were the geographical areas covered by the programme? What are the reasons these areas were chosen?

b) Where there any geographical areas excluded from the programme? What were the reasons for this exclusion? Please elaborate your answer.

5-a) What are/were the target groups within the HIV prevention programme and what are the reasons these groups were chosen?

b) Were there any difficulties encountered when reaching these target groups?

6-a) In your opinion, what are/were the challenges/obstacles that the programme is/was facing in general? What were/are the promoting and aiding factors facilitating the programme? Can you please elaborate your answer?

b) What were/are the specific challenges you faced with your target group or specialized unit of the prevention programme?

c) Over its course, how have the challenges facing the HIV prevention programme changed?

7-In your opinion, are/were there any cultural/social challenges in the community work within the HIV prevention programme? If yes, please elaborate and give an example from your experience. If no, please explain how the programme has not been affected by any social/cultural challenges.

8-How were the challenges faced by the programme resolved? What do you think can be further done to overcome these obstacles the programme faced?

9-a) Tell us what you think about the implementation of the programme. Do you feel the programme was implemented as planned?

b) If no, what in your opinion was different from the original plan? Can you please explain?

c) What kind of problems were encountered during implementation? Please elaborate your answer.

d) What were the specific obstacles of implementation within your specialized unit of the prevention programme? Please explain.

10-Can you tell us from your perspective, what do you think the negative and positive effects of the HIV prevention programme during its course? Can you elaborate your point of view?

11-On a scale from 0 (total failure) to 10 (total success), where would you place the success of the programme? Please give reasons for your choice.

**References**

1. UNAIDS. Middle East and North Africa:Regional Report on AIDS 2011.Geneva: UNAIDS- Joint United Nations Programme on HIV/AIDS; 2011.
2. Federal Ministry of Health. Global AIDS Response Progress Reporting 2012-2013. Sudan: UNAIDS- Joint United Nations Programme on HIV/AIDS; 2014.
3. UNAIDS. Sudan Country factsheet 2017, [Cited 2017 Sep 22]. Available from: <http://www.unaids.org/en/regionscountries/countries/sudan>
4. Ministry of Public Health and Population. Country Progress Report 2013 Yemen. Yemen: UNAIDS- Joint United Nations Programme on HIV/AIDS; 2014.
